# Epilepsy and quality of life in Iranian epileptic patients

**DOI:** 10.1186/s41687-021-00292-3

**Published:** 2021-01-28

**Authors:** Bahareh Honari, Seyed Mehran Homam, Maryam Nabipour, Zahra Mostafavian, Arezou Farajpour, Nyusha Sahbaie

**Affiliations:** 1 Faculty of Psychology and Educational Sciences, Semnan University, Semnan, Iran; 2 Department of Neurology, Mashhad Branch, Islamic Azad University, Mashhad, Iran; 3 Faculty of Medicine, Mashhad Branch, Islamic Azad University, Mashhad, Iran; 4 Department of Community Medicine, Mashhad Branch, Islamic Azad University, Mashhad, Iran; 5 Department of Education Development Center, Mashhad Branch, Islamic Azad University, Mashhad, Iran

**Keywords:** Epilepsy, QOLIE-31 questionnaire, Quality of life

## Abstract

**Background:**

Epilepsy is one of the most common neurological disorders with physical, emotional, and social consequences. Previous studies indicate that epilepsy symptoms can highly affect the epileptic patients’ satisfaction in life. The aim of the present study is to investigate the QOL of People with Epilepsy (PWE) in Khorasan Razavi province, Iran.

**Methods:**

In this study, 100 patients were randomly selected. After confirmation of the diagnosis of epilepsy by neurologists and fulfilling the entrance criteria, patients completed the Quality of Life in Epilepsy-31 inventory (QOLIE-31) questionnaire. Finally, data was analyzed statistically by SPSS software.

**Results:**

The study sample comprised 100 PWE, aged 18–74 years (34 ± 13), of whom 58 (58%) were females. Tonic-colonic seizure was the most common (60%) type of seizure. The obtained score of each subscale and the range of the QOLIE-31 total score was 16.40–79.18 with the mean of 50 (SD = 16). The energy-fatigue subscale score was significantly higher in patients younger than 35 (*p* = 0.018). The data analysis showed that the seizure worry subscale was significantly higher in single patients (*p* = 0.04). Duration of epilepsy had a positive correlation with QOLIE-31 total score (*p* = 0.038), and a negative relationship with energy-fatigue subscale (*p* = 0.018). In contrast with previous studies, which reported the frequency of the epileptic episodes as the most important predictor of QOL, our results showed no significant correlation between the number of the episodes and overall QOL score (*p* = 0.063). However, the number of episodes was significantly correlated with emotional well-being and cognition subscales. Furthermore, the results indicated that poor QOL score is correlated with depressed mood.

**Conclusion:**

In fact, the ultimate and preferred outcome of all treatments and care interventions is the patient’s QOL. Thus, improvement of the QOL by means of obtaining more information about its contributing factors, in PWE should be one of the main goals in the patients’ treatment.

## Background

Epilepsy is a serious brain disorder in which it has different manifestations such as different types of seizures and syndromes [1].

A meta-analysis done in Iran has showed that epilepsy prevalence is 5%, and it was estimated that 3% of patients are younger than 20 years of age [2].

The World Health Organization (WHO) defined Quality of Life (QOL) as the conception of individuals’ of their position in life which is framed by culture and its value system [3]. As epilepsy is a chronic illness and has a nature of epileptic seizures, it can have multiple effects on a patient’s perception about quality of and satisfaction with life [4]. Moreover, epilepsy can be associated with physical, emotional, and social outcomes [5].

In previous studies, epileptic patients have reported a low QOL because they may have lower self-esteem, and greater anxiety, and depression. In some patients, social stigma and its impacts on QOL lead to a challenge especially in severe clinical manifestations [6]. It is believed that demographic factors also play a crucial role in QOL [7].

There are so many tools that have been used to measure the QOL in epileptic patients, some of these tools are for general use and some are designed specifically for epileptic patients, such as the quality of life in epilepsy- 31 inventory (QOLIE-31) questionnaire [8].

QOLIE-31 is one of the most accepted tools, which is used especially in adults 18 and older. This tool, in fact, is derived from QOLIE-89. QOLIE-31 has been used in multiple clinical studies as a standard tool. The reliability and validity of the Persian version of this tool has been evaluated in Iran [9].

Although 80% of People with Epilepsy (PWE) live in developing countries, there’s little information about patients’ QOL in low-income countries [10]. A systemic review of QOL in 2011 showed that 61 of 86 studies in this field had been done in Europe and North America, developed countries with high income [11]. So far, our knowledge about the quality of life in Iranian epileptic patients has been obtained from the short form 36 health survey questionnaire (SF-36) [12, 13].

Since there are a few literatures about the evaluation of QOL in Iranian PWE, This study aims to identify the effect of demographic and disease features on epileptic patients’ QOL in Iranian society by means of QOLIE-31 questionnaire.

## Material and methods

The current study has been performed on the epileptic patient members of Khorasan Razavi Epilepsy Society in 2018. Most of the epileptic patients in the east of Iran are members of this society.

The sampling method was random. The sample size was measured based on the study of Mohammadi et al. [14]. The following formula was used to calculate the sample size:

$$ n={\left(\frac{z1-\propto \times \partial }{d}\right)}^2 $$

One hundred and ten patients entered our study. Inclusion criteria include having medical records and diagnosis of epilepsy according to a neurologist’s opinion, at least two epileptic episodes in the last 12 months, passing at least 1 year from the onset of the disease, age 18 and older, receiving anti-epileptic drugs, and the ability to provide written consent. None of the patients were in hospitals when they were fulfilling the questionnaire.

Exclusion criteria include age 66 and older, non-epileptic seizures, seizures caused by drug abuse or medications or other emergency situations, inappropriate psychological conditions, chronic underlying disease, having an incurable disease, and unsolicited personal consent.

### Materials

In this study we used the Persian version of QOLIE-31 questionnaire and a checklist which contained demographic variables (age, sex, employment status, marital status) and information about the disease (the nature of the episodes, duration of disease, number of episodes per month, familial history of epilepsy and history of hospitalization because of the disease). QOLIE-31 is a questionnaire that consists of 31 items and 7 subscales. They are emotional well-being, seizure worry, overall QOL, social function, medication effects, cognitive function, and energy/fatigue [15].

Patients’ answers to each question are provided to polytomous response options. Scores range from 0 to 100, and a higher score shows better QOL [16]. Scores calculated as T scores.

## Methods

In this study, 115 patients were selected from Khorasan Razavi epilepsy community. Three patients because of the long-distance, one patient because of the age 66 and older, three patients because of chronic disease, one patient because of the dissatisfaction with the company and, two patients because of the age under 18 were excluded from the study. One hundred and five patients participated in the study. Five forms that we received were incomplete. Eventually, One hundred forms were analyzed. After controlling the inclusion and exclusion criteria, a neurologist’s visit and confirmed the diagnosis of epilepsy patients entered the study. After the researcher’s explanation about the study and obtaining informed consent, patients filled out the questionnaires. None of the patients were in hospital when they completed the questionnaire.

### Ethical considerations

People in this study were patients that were willing to participate. There was no necessity to write the names in the questionnaire, and all the information was confidential. Research plans were ratified in the ethic committee of Mashhad Islamic Azad Medical University.

### Data analysis

Data were analyzed using software SPSSv20. We report means, standard deviation, and percentage ratio. Student’s t-test was used to compare the overall score of QOL and subscales between two groups. Analysis of variance was used in more than two groups, and the Pearson product-moment correlation coefficient was used to study the correlation between duration of disease and number of episodes per month with the overall score of QOL and subscales.

Linear regression test was also used to study independent effects of variables, age and duration of disease on subscales, and energy/fatigue. A significant level of 5% was used.

## Results

### Demographic and clinical statistics

Responsiveness level of participants was 96%. The age range of patients was 18–74 with a mean age of about 34 (SD = 13). Fifty-eight participants (58%) were female. Demographic features of patients are shown in Table 1, and clinical features are listed in Table 2.

**Table 1:** Demographic characteristics of the study subjects

Demographic Characteristics		N (%)
**Sex**	Female	58 (58%)
	Male	42 (42%)
**Marital status**	Married	57 (57%)
	Single	43 (43%)
**Education**	Illiterate	2 (2%)
	Elementary	27 (27%)
	High School Diploma, Bachelor Degree	66 (66%)
	Master Degree, PhD	5 (5%)
**Occupation**	Governmental	15 (15%)
	Non-Governmental	27 (27%)
	Jobless	58 (58%)
**Age (in years)**	Range = 18–74 mean = 34 SD = 13

**Table 2:** Clinical characteristics of the study subjects

Clinical characteristics		N (%)
**Type**	Tonic-clonic	60 (60%)
	Absence	3 (3%)
	Myoclonic	3 (3%)
	Tonic	12 (12%)
	Clonic	0 (0%)
	Atonic	5 (5%)
	Partial	17 (17%)
**Family history**	Yes	31 (31%)
	No	69 (69%)
**Hospitalization due to Epilepsy side effects (lifetime)**	Yes	23 (23%)
	No	77 (77%)
**Duration of the disease (year)**	Range = 1–56 mean = 13.4 SD = 11.2	
**Number of seizure episodes per month**		**N (%)**
	1	14 (14%)
	2	16 (16%)
	3	41 (41%)
	4	13 (13%)
	5	16 (16%)

Statistical data about clinical features show that 60% of our patients have tonic-colonic epilepsy. Sixty-nine percent of patients did not have a positive familial history, and 23% were hospitalized because of episodes or their complications. The average duration of the disease is 13.4 (SD = 11.2) years, and the average number of episodes is 3 ± 1.2 times in a month.

### Quality of life

In this study, the QOLIE-31 overall score ranged from 16.4 to 79.2, and the average overall score was 50 (SD = 16). Table 3 shows descriptive statistics about the QOLIE-31 score with its subscales separately.

**Table 3:** Descriptive statistics in quality of life in epilepsy-31 inventory and Pearson correlation of sub-scales with overall score

Scale	Mean	SD	Pearson correlation with overall score (P)
Seizure worry	40.8	32.1	*r* = 0. 53 (*p* = 0.000)
Overall quality of life	30.9	14.2	*r* = 0. 59 (*p* = 0.000)
Emotional well-being	48.1	21.9	*r* = 0. 72 (*p* = 0.000)
Energy/fatigue	48.2	20.6	*r* = 0. 57 (*p* = 0.000)
Cognitive functioning	57.4	25.2	*r* = 0. 76 (*p* = 0.000)
Medication effects	51.6	25.9	*r* = 0. 55 (*p* = 0.000)
Social functioning	56.7	23.3	*r* = 0. 82 (*p* = 0.000)
Overall score	49.7	16	

There was no significant statistical relationship between QOLIE-31 overall score and clinical and demographic variables such as gender, education, and occupational status.

According to Table 4, patients were divided into two groups of over 35 years of age (*n* = 34) and younger than 35 (*n* = 66). The energy/fatigue subscale was significantly higher in the group under 35. (*p* = 0.018).

**Table 4:** QOLIE-31 subscales based on age groups

subscale	Age (inyears)	Mean	Std. Deviation	t	***p***-value
Seizure worry	< 35	42.8118	32.64781	.869	.387
	> 35	36.9094	31.23813	.881	.381
Overall quality of life	< 35	31.2500	14.90773	.331	.742
	> 35	30.2500	13.10173	.345	.731
Emotional well-being	< 35	49.2121	22.53989	.673	.502
	> 35	46.0606	20.71524	.693	.491
Energy/fatigue	< 35	51.7424	19.18518	2.411	.018
	> 35	41.4706	22.00308	2.307	.025
Cognitive functioning	< 35	56.6760	25.22070	−.392	.696
	> 35	58.7833	25.58253	−.390	.698
Medication effects	< 35	51.9338	27.60702	.174	.862
	> 35	50.9765	22.62678	.186	.853
Social functioning	< 35	59.6308	23.45979	1.723	.088
	> 35	51.2059	22.41182	1.748	.085
Overall score	< 35	50.7708	16.33256	.880	.381
	> 35	47.7377	15.43315	.896	.373

Seizure worry subscale score had a significant statistical difference (*p* = 0.04) between the married group (35.09 + 30.4) and single group (48.3 + 33.1).

The Pearson correlation coefficient showed a positive statistical correlation between duration of epilepsy and the QOL overall score (*r* = − 0.219, *p* = 0.038) and a negative relationship with energy/fatigue subscale (*r* = − 0.244, *p* = 0.018).

There was no significant association between the number of episodes and QOL overall score (*P* = 0.063). However, there were statistically significant positive correlations of frequency of epileptic seizures per month with emotional well-being (*r* = 0.202, *p* = 0.045) and cognitive functioning (*r* = 0.236, *p* = 0.019).

Linear regression analysis was done to assess the effect of age and duration of epilepsy on the fatigue/energy subscale. Only age had significant relationship with fatigue/energy subscale (*r* = − 0.3, *p* = 0.004).

Emotional well-being is evaluated by means of five questions (questions number 3,4,5,7 and 9) in QOLIE-31 questionnaire. As the Table 5 shows, the score of all five questions related to the emotional well-being have a significant positive correlation with QOL total score.

**Table 5:** Correlation of emotional well-being related question with total QOL score

	Question 3	Question 4	Question 5	Question 7	Question 9
**Correlation Coefficient**	.595	.522	.463	.551	.535
***P*** **value**	.0001	.0001	.0001	.0001	.0001

## Discussion

In this study, we evaluated the QOL of 100 epileptic patients in the east of Iran who are covered by the Khorasan Razavi Epilepsy Association.

### Demographic features

Surprisingly, our findings showed that there was no significant relationship between QOL score and demographic features such as sex, employment, and education level, while epidemiologic studies on evaluation of QOL related factors in Iran suggested that poor economic status and lower education level are the most important predictors of lower QOL in Iranian society [17–19]. Also, a study in western Iran showed that socioeconomic status, physical inactivity, chronic diseases and lack of health insurance coverage are the most influential factors that cause poor QOL [18]. A previous study has reported a significant negative correlation between female gender and lower educational level with the QOLIE score in epileptic patients. Furthermore, this study represented that the majority of epileptic patients are not employed, although this factor had no significant effect on our patients’ quality of life score [20]. The differences between our finding and earlier projects might be because of smaller sample size in the present study.

Considering the given data in Table 4, we can infer that patients younger than 35 years of age achieved higher energy/fatigue score. A study by Djibuti revealed that age strongly correlates with overall quality of life, energy/fatigue, and cognitive scores [20].

According to the data, married patients had a lower seizure worry subscale score. A study by Tajvar et al. in 2008 showed a better QOL in married individuals in Iran [17]. Also, a study done by Zhao et al. reported that married PWE achieved a higher QOLIE-89 total score [21]. The decreased seizure worry subscale score of the married PWE group in our study may be due to impaired social support in Iranian society [22].

### Quality of life in epileptic patients

In the present study, the overall QOL score is 50 (SD = 16). A study done by Saadi et.al, compared the total QOLIE score globally. They reported that Russia and Canada with 42.1 and 82 scores had the lowest and highest recorded scores respectively [23]. Consistent with our results, previous studies have showed that the duration of epilepsy disease has a negative relationship with the energy- fatigue score [24]. Moreover, we revealed that there is a positive correlation between disease duration and the total QOL score. Szaflarski et.al also reported the same result. They believed that the coping mechanism and achieving an optimal treatment due to multiple medication trials can lead to a better QOL in patients with longer duration of epilepsy disease [25]. In contrast to previous reports, which indicated that the frequency of seizure episodes is the most important predictor of poor QOL in epileptic patients [24, 26, 27], our results showed no significant relationship between the number of episodes and total QOLIE-31 score. However, in this study, the frequency of episodes was negatively correlated with emotional well-being and cognition subscales. Geukht et.al suggested that since the frequent episodes of seizure interferes with routine daily activity of PWE, it would dramatically affect the QOL [24].

According to the data of Table 5, the scores of the five questions related to the emotional well-being subscale were positively correlated with QOL total score. The mood of patients can be evaluated using the results of those five questions which determine emotional well-being. Base on this fact, we can infer that the depression is one of the determinants of poor QOL which is supported by previous studies [28, 29].

Patients are often the best resource of information. Medical technology can evaluate the physical, physiological, and biomedical data, but these data cannot array comprehensive images about the patients’ situation or treatment results. Therefore, a lot of information can be available only from patients [30, 31].

Patients-reported outcomes can be used to evaluate medical treatment successes. Conception and acceptance of health and disease status depends on social status. Social status such as socioeconomic level and race, have an important role in treatment and its results [32].

Main social factors affecting health are known as social determinants of health; situations in which people were born, grew up, or lived. Social determinants of health also affect individual and social results by biological factors, and they are an important cause of health inequality. Socioeconomic status is about social status and hope for living according to education, income, and occupation [33, 34].

Studies show that social and economic deprivation increases the prevalence and incidence of epilepsy. Epileptic patients have lower education, income, and health conditions. Finding a job is also more difficult for them. Other studies reported relations between failures to follow treatment, low socio-economic level, and unsecured insurance coverage in epileptic patients. Housing, education, employment status, and nutrition are social determinants of health that mediate epilepsy care and results. People who live in high standard areas with better quality health care receive better treatment results [34, 35].

Epileptic patients are more in danger of psychological complications compared to the general population. Studies in the last decade show that psychological treatment has a great impact on the patients’ QOL [36].

While 80% of epileptic patients live in developing countries, the primary goals of treatment are focused on physical complications and the control of seizures, while epileptic patients suffer from several psychological challenges such as low self-confidence, depression, and social challenges such as driving constraint, unemployment, and social isolation. These patients are less likely to get married, and they are more likely to get a divorce. Although epilepsy has effects on different social dimensions, marriage is the first source of social support and predicts health status. Studies show that married people report better psychological and physical health compared to single people [37].

It is obvious that QOLIE-31 overall score is under the influence of cultural, social, and economic factors. Higher QOLIE-31 score in a country represents better information about the disease, decrease in stigma, social support and patients’ education and awareness about the disease and self-care. A lower score, such as those in this study and in Bhutanese [38] indicates that more education and awareness about the disease is necessary. Therefore, it seems that further QOL related studies are needed in developing countries.

### Limitations

The regional focus on one province of Iran (Khorasan Razavi) and the small number of individuals can be considered as limitations of this study, which requires more cross-sectional studies in the future.

## Conclusion

In the present study we have identified that PWE younger than 35 years of age had higher energy-fatigue subscale scores. Moreover, the data indicates that the duration of epilepsy had a positive correlation with QOLIE-31 total score. Also, In contrast with previous studies, our results showed no significant correlation between the number of the episodes and overall QOL score. Unlike the earlier projects, single individuals obtained better seizure worry subscale scores compared to married patients in the present study. Supported by prior studies, our statistical analysis showed that depressed mood was correlated with poor QOL. As the QOL in PWE can be considered as a reflection of cultural, social, and disease-related factors, we suggest that it is up to health care system to give information and educate PWE and the society about the possible overwhelming factors which may affect the QOL in epileptic patients. This issue helps the society and the PWE to achieve a better understanding of the challenges an epileptic patient is dealing with.

## Data Availability

The datasets used and analyzed during the current study are available from the corresponding author on reasonable request.

## Abbreviations

QOL: Quality of Life
PWE: Patients with Epilepsy
WHO: World Health Organization
QOLIE-31: Quality of Life in Epilepsy- 31 inventory
